# DADOS-Prospective: an open source application for Web-based prospective data collection

**DOI:** 10.1186/1751-0473-1-7

**Published:** 2006-11-13

**Authors:** Lam Nguyen, Anand Shah, Matthew Harker, Henrique Martins, Mariana McCready, Andreia Menezes, Danny O Jacobs, Ricardo Pietrobon

**Affiliations:** 1 School of Medicine, University of North Carolina-Chapel Hill, Chapel Hill, NC 27599, USA; 2 School of Medicine, University of Pennsylvania, 3450 Hamilton Walk, Philadelphia, PA, 19104, USA; 3 Center for Excellence in Surgical Outcome, Division of Orthopaedic Surgery, Duke University Medical Center, Box 3094, Durham, NC, 27710, USA; 4 Department of Surgery, Duke University Medical Center, Box 3704, Durham, NC, 27710, USA; 5 Division of Orthopaedic Surgery, Department of Surgery, Duke University Medical Center, Durham, NC, 27710, USA

## Abstract

**Background:**

Randomized, prospective trials involving multi-institutional collaboration have become a central part of clinical and translational research. However, data management and coordination of multi-center studies is a complex process that involves developing systems for data collection and quality control, tracking data queries and resolutions, as well as developing communication procedures. We describe DADOS-Prospective, an open-source Web-based application for collecting and managing prospective data on human subjects for clinical and translational trials. DADOS-Prospective not only permits users to create new clinical research forms (CRF) and supports electronic signatures, but also offers the advantage of containing, in a single environment, raw research data in downloadable spreadsheet format, source documentation and regulatory files stored in PDF format, and audit trails.

**Results:**

Feedback from formal and field usability tests was used to guide the design and development of DADOS-Prospective. To date, DADOS-Prospective has been implemented in five prospective clinical studies at our institution. Four of these studies are still in the CRF creation phase and one study has been entirely launched.

**Conclusion:**

DADOS-Prospective has significant advantages over existing Web-based data collecting programs. At our institution, it has been demonstrated to be an efficient tool for prospective clinical studies.

## Background

Randomized, prospective trials involving multi-institutional collaboration have become a central part of clinical and translational research. However, data management and coordination of multi-center studies is a complex process that involves developing systems for data collection and quality control, tracking data queries and resolutions, as well as developing communication procedures, to name a few. Such cumbersome processes have deemed traditional, paper-based data collection and management systems increasingly less efficient. More recently, the Internet has arisen as an efficient and secure tool for collecting and managing information in single-site and multi-site prospective clinical trials and observational studies involving registries [1].

Advances in Web-based data collection have allowed for remote data entry through clinical research forms (CRF) that can be accessed on the Internet from anywhere. Since information can be collected from different locations and stored into a secure central database, the Internet facilitates the onerous task of data quality control involved in multi-institutional studies, allowing for real-time centralized data monitoring and auditing. Furthermore, compared to the traditional paper-based method, Web-based data entry offers the advantage of faster availability of data for sharing, processing and analysis, and, importantly, greater security for data storage and archiving [2].

Commercial products have greatly evolved over the years to maximize efficiency of Web-based data collection in studies involving multiple centers and large data registries. However, some of these commercial tools can be expensive, difficult to use, and may require considerable customization in order to implement [3]. In this article, we describe DADOS-Prospective, an open-source Web-based application for collecting and managing data on human subjects for clinical and translational trials. DADOS-Prospective not only permits users to securely archive and download data and to create new CRFs, but also offers the advantage of containing, in a single environment, CRFs, raw research data in regular spreadsheet format, source documentation and regulatory files stored in PDF format, and audit trails. The combination of these factors in a single location greatly streamlines the research process, allowing for efficient data collection and study management. We outline below the design objectives, software architecture, implementation, usability, and future directions for DADOS-Prospective.

## Methods

### Design objectives

In designing the DADOS-Prospective application, our primary objective was to build a Web-based tool for data collection on human subjects for clinical and translational trials. Furthermore, we required that the application's data collection tool achieve full compliance with Title 21 Code of Federal Regulations (21 CFR Part 11) for Electronic Records and Electronic Signatures and HIPAA guidelines for storing and collecting patient data on secure database[4]. Specifically, DADOS-Prospective was to completely integrate 21 CFR Part 11 and HIPAA guidelines by satisfying the following requirements:

1. Support electronic signatures.

2. Have a trail record for changes made to the data entry once the form has been signed

3. Be maintained in a secure protocol such as HTTPS

4. Allow for storage of source documentation in PDF (portable document format) attached to each subject record in order to facilitate audits performed remotely.

Within the framework of these requirements, we specifically outlined several technical features that are highly desirable for DADOS-Prospective. Before developing the application, we analyzed similar tools, including commercially available products, and recommendations from HIPAA (Health Privacy and Accountability Act) and Title 21 Code of Federal Regulations (21 CFR Part 11) for Electronic Records and Electronic Signatures (FDA, 2005) [4]. Our search resulted in a series of technical objectives for the program:

1. The application should be able to create clinical research forms (CRFs) based on a templating system that takes previous CRFs as their model.

2. CRFs should allow for any type of fields (dates, pictures, text, etc) and these fields should be arranged in any table format.

3. The application should be able to contain non-traditional fields such as upload of electronic fields and image files with immediate display (for clinical images such as x-rays, computerized tomography, etc)

4. CRFs should be easy to modify once created, but CRFs should not be modified once data have been entered. The latter feature preserves data integrity.

5. Data extraction should be simple and easy to use, providing all the data required by data analysts in a spreadsheet format.

6. DADOS-Prospective should serve as a study management system, containing the capability to support future interview scheduling, and the ability to add/update patient information and participating users in a simple process.

7. Contain, in a single location, the audit trail, data in a regular spreadsheet format, and source documentation in PDF (portable document format). The combination of these three factors allows for easy auditing of the application.

8. The technology should be user-friendly so that users can create and modify CRFs and enter data directly on the interface, reducing the number of pop-up windows and the number of clicks needed to navigate through the application and save information.

### Software architecture

**Figure 1:**
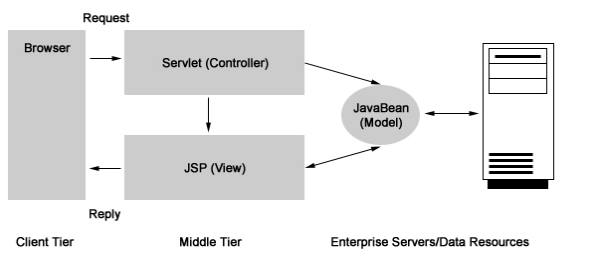
**Model-View-Controller (MVC) design paradigm**. [Adapted from Pietrobon R, Shah A, Kuo P, Harker M, McCready M, Butler C, Martins H, Moorman CT, Jacobs DO: Duke Surgery Research Central: an open-source Web application for the improvement of compliance with research regulation. BMC Med Inform Decis Mak 2006, 6(1): 32]

The software was modeled after other software applications designed by our group using JAVA as the programming language, MVC2 as the design model and, because of the need of achieving functionality for feedback and system growth, the evolutionary prototyping was used [5]. The idea behind the Model-View-Controller design model (MVC) is that an application consists of three critical components: a Model, Views of the Model, and Controllers. The Model is the part of the application that contains the actual application logic. The Model facilitates database access, computes numbers, and manipulates data structures. The View and Controller represent the user interface of the application. The user interface is conceptually split into input and output components. The Controller is an input component that supplies information to the Model. The View is an output component which displays information from the Model. The View typically communicates with the Model by registering itself as a callback and responding to events generated by the Model. [see Figure 1, adapted from Pietrobon R, Shah A, Kuo P, Harker M, McCready M, Butler C, Martins H, Moorman CT, Jacobs DO: Duke Surgery Research Central: an open-source Web application for the improvement of compliance with research regulation. BMC Med Inform Decis Mak 2006, 6(1): 32].

### Implementation

There are three different levels of access to DADOS-Prospective: administrator, coordinator, and interviewer. The administrator, typically a Webmaster, has total access to all projects enrolled in the application, including the ability to modify the content of any study that has not been launched. The administrator has exclusive rights to extract research data for analysis at any time. Coordinators may create new studies, access and modify forms for only those studies that belong to them. As in the case of administrator access, coordinators may not modify the content of a study for which data entry has been initiated; this feature serves to protect data integrity. Interviewer access is strictly limited to data entry.

**Figure 2:**
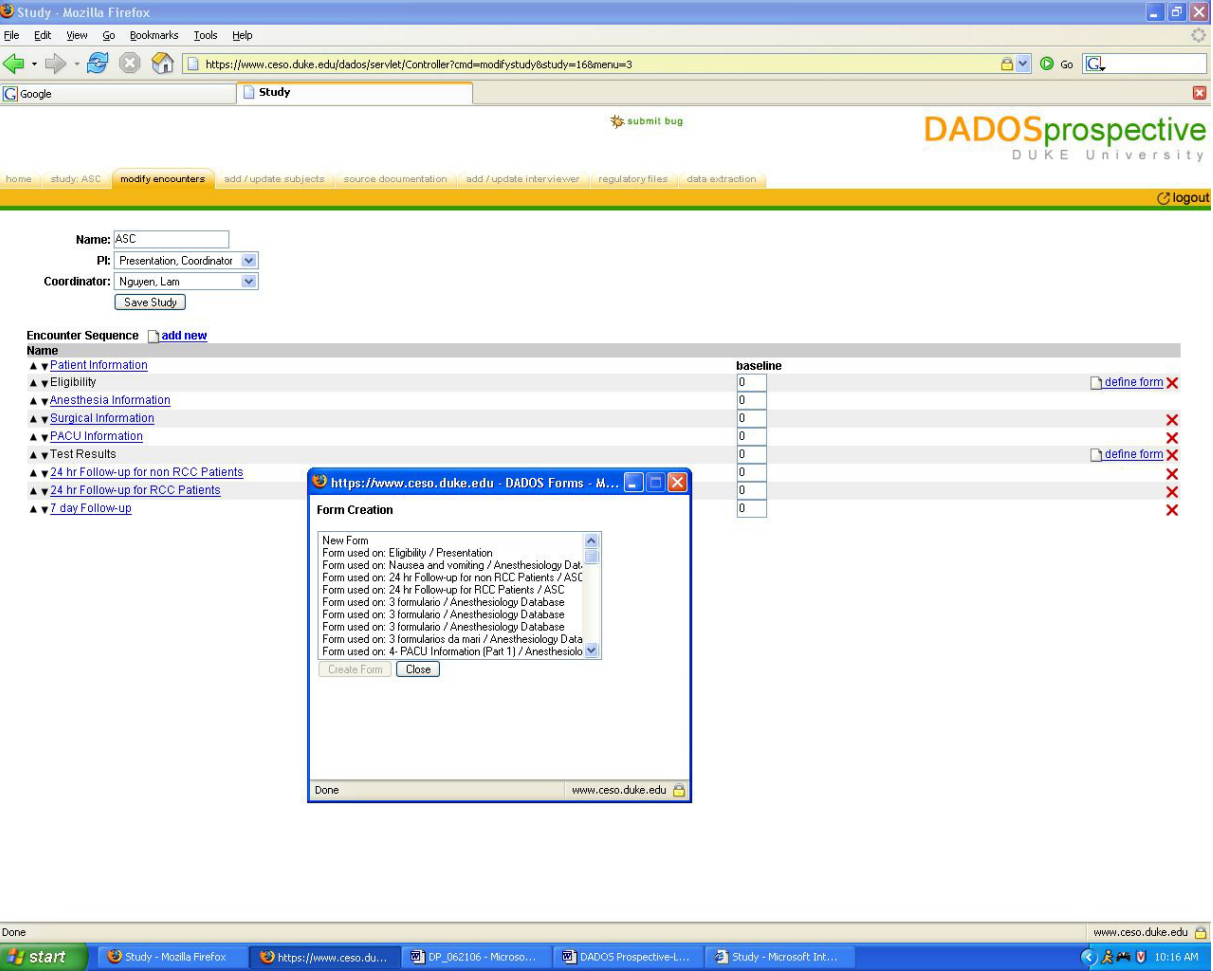
**Navigational toolbar**. Encounter creation interface depicting dropdown menu from which a coordinator may choose an existing form to modify for a new study, or create an entirely new form.

Each principal investigator (PI) is initially given access to the DADOS-Prospective website as a coordinator by using a login ID and password. Once authenticated into the application, the PI may create a new study and view a list of projects he/she is currently conducting. When a new project is created, different roles can be assigned (i.e. PI, coordinator, interviewer). Once assigned as a PI, the user may no longer create or modify forms. Only the coordinator and the administrator can create and modify the content of a study. The coordinator may modify a study assigned to them by selecting its name and using the toolbar at the top of the page, as shown in Figure 2. Each of the options in the toolbar is explained in detail in Table 1.

**Table 1:** Navigation toolbar. Functions for each of the menu items found on the DADOS-Prospective navigation toolbar.

Home	Return to the list of all existing projects to which user is allowed access
Study: (name of study)	View a list of all subjects enrolled in the selected study, or search for subjects enrolled in the selected study by "Name," "MRN," or "DOS."
Modify Encounters	Create/modify encounters and their associated baseline and CRFs
Add/Update Subject	Enroll a new or an existing subject into the selected study, or delete a subject from the study. Add new subjects to the database without enrolling them into the selected study, or delete them from the database.
Source Documentation	Add/upload or delete any reference documentations pertinent to the study (i.e. rating scales, codes)
Add/Update Interviewer	Add to or delete from the study personnel with data entry access only.
Regulatory File	Upload or delete regulatory files related to the study
Data Extraction	Download the study's data in Excel spreadsheet format
New User	Add new users who may access the study as a coordinator or interviewer

When creating a new study, the coordinator may utilize the navigation toolbar described in Table 1 to setup the study's content. Choosing the tab labeled "modify encounter," the coordinator may create the different encounters (i.e. Eligibility, Test Results, Treatment 1) for the study. Once all encounters are created, they may be arranged in a desired sequence by using the up/down arrows next to each encounter to move them up or down the list [see Figure 2]. Note, the software defines "baseline" as the day of the first encounter with a patient, thus the encounter on top of the list is automatically defined as the baseline encounter [see Figure 2]. For subsequent encounters, all numerical values entered into the boxes underneath baseline will be interpreted as the number of weeks until the next encounter is to be executed, counting from the date on which the baseline encounter (the very first encounter on the list) is completed. With this feature, the program calculates the exact date on which an encounter is to take place, giving the users easy access to all future interview scheduling. Simply clicking on a patient's name will display all previously defined encounters and the date when each encounter will occur.

**Figure 3:**
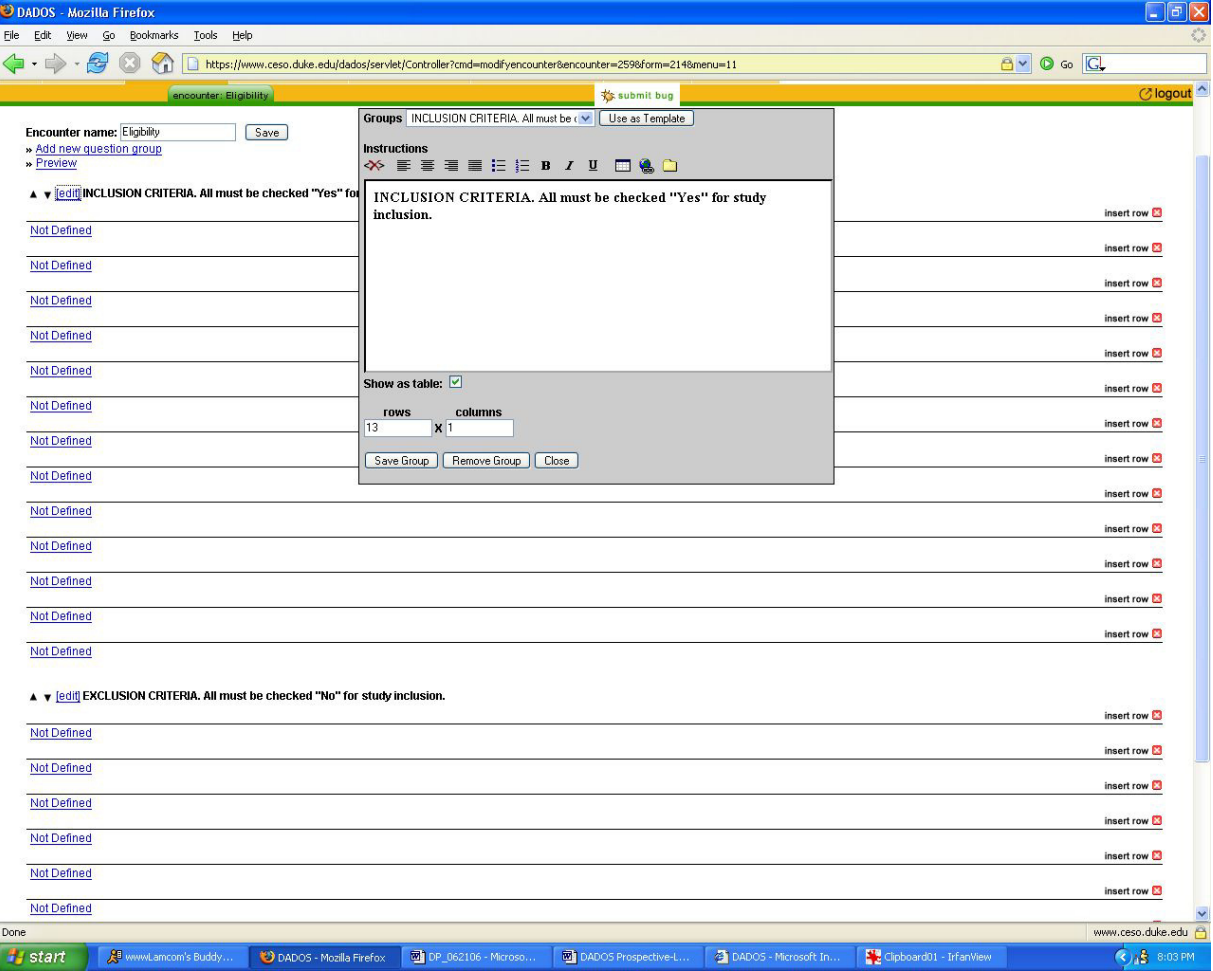
**Form creation**. Form creation interface depicting how a coordinator can define a CRF in any table format.

**Figure 4:**
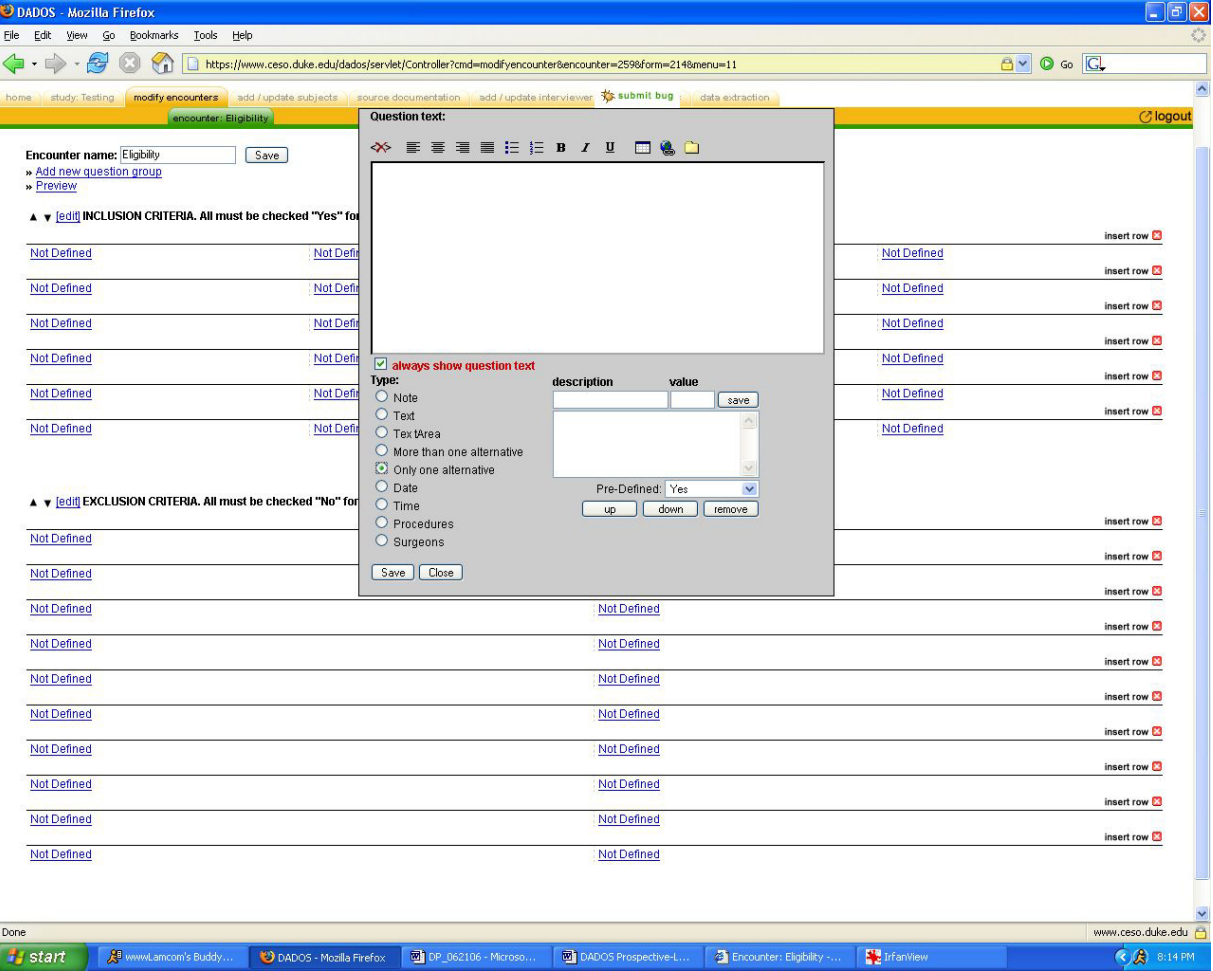
**Form configuration**. Form configuration interface that demonstrates how a coordinator can define any field in the form to specific types, such as text, dates, pictures, etc.

The coordinator can define a CRF for each encounter by selecting "define form." He/she has the option to select an existing form to edit or use "as is" that has been created in the system from previous studies, or the user can choose to create a new form which can be customized to fit his/her particular study [see Figure 2]. Once the new form is created, it is then saved and added to the list of existing forms that can be used as a template for creating future forms. DADOS-Prospective has a mechanism for creating CRFs based on a templating system that takes previous CRFs as their model. The application offers a unique feature that allows the investigator to customize the CRF to any table format [see Figure 3]. Furthermore, the coordinator may define any field in the form to specific types, such as text, dates, pictures, etc [see Figure 4]. The program also supports non-traditional fields that can add Internet links and upload electronic files, including most image and video formats. This feature is highly desirable for investigators who wish to create questions that require subjects to view an external Web link or an image file such as an x-ray. Once customization is complete, selecting the "save" button applies the changes to the form. All forms can be continually modified and improved upon as long as data has not been entered. Only the updated version of the form is kept in the database to avoid confusion of multiple versions. Once data collection has begun, users may no longer edit the forms; this feature preserves data integrity.

**Figure 5:**
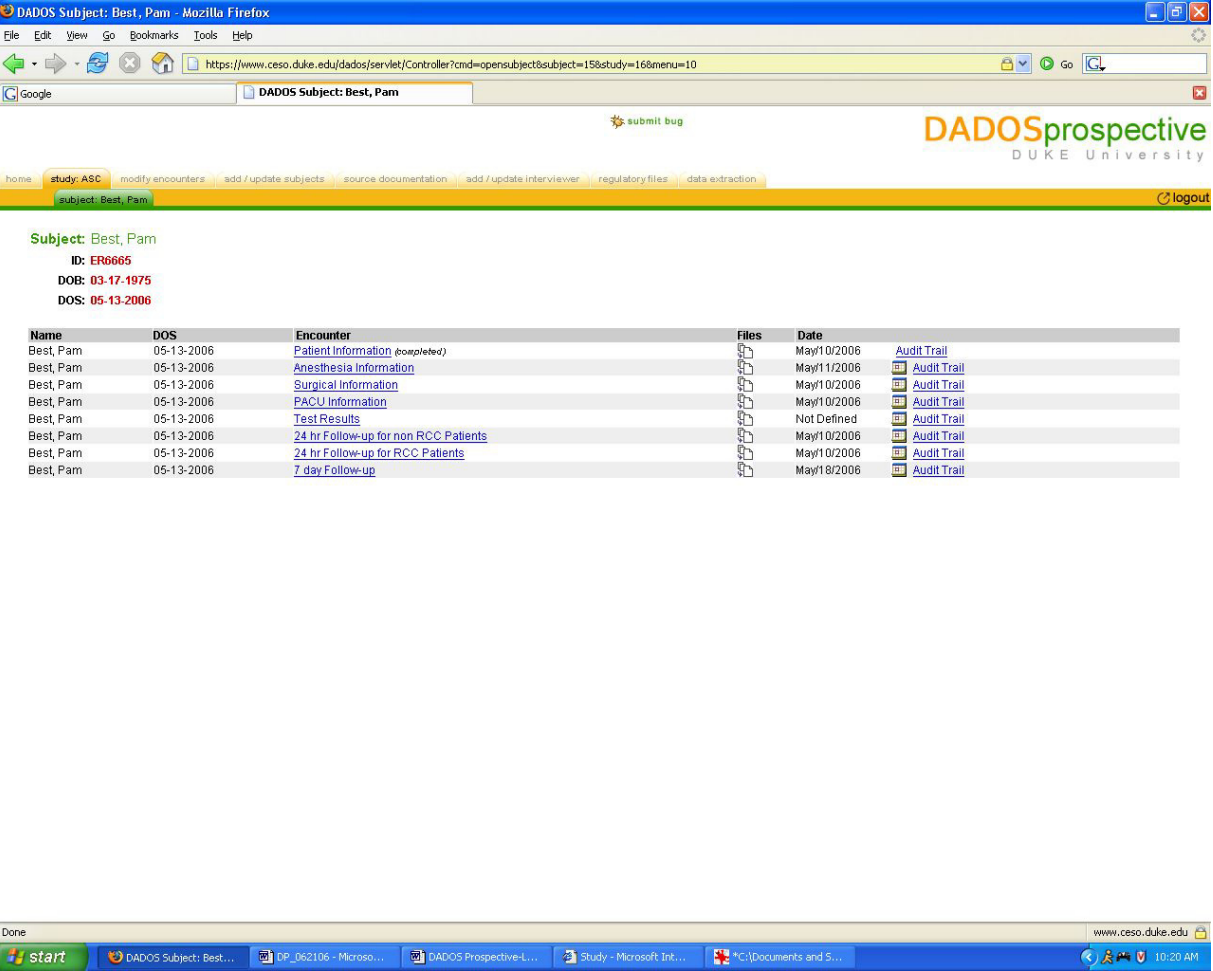
**Subject interface**. Interface depicting defined encounters, interview scheduling and data audit trails for the selected study participant.

**Figure 6:**
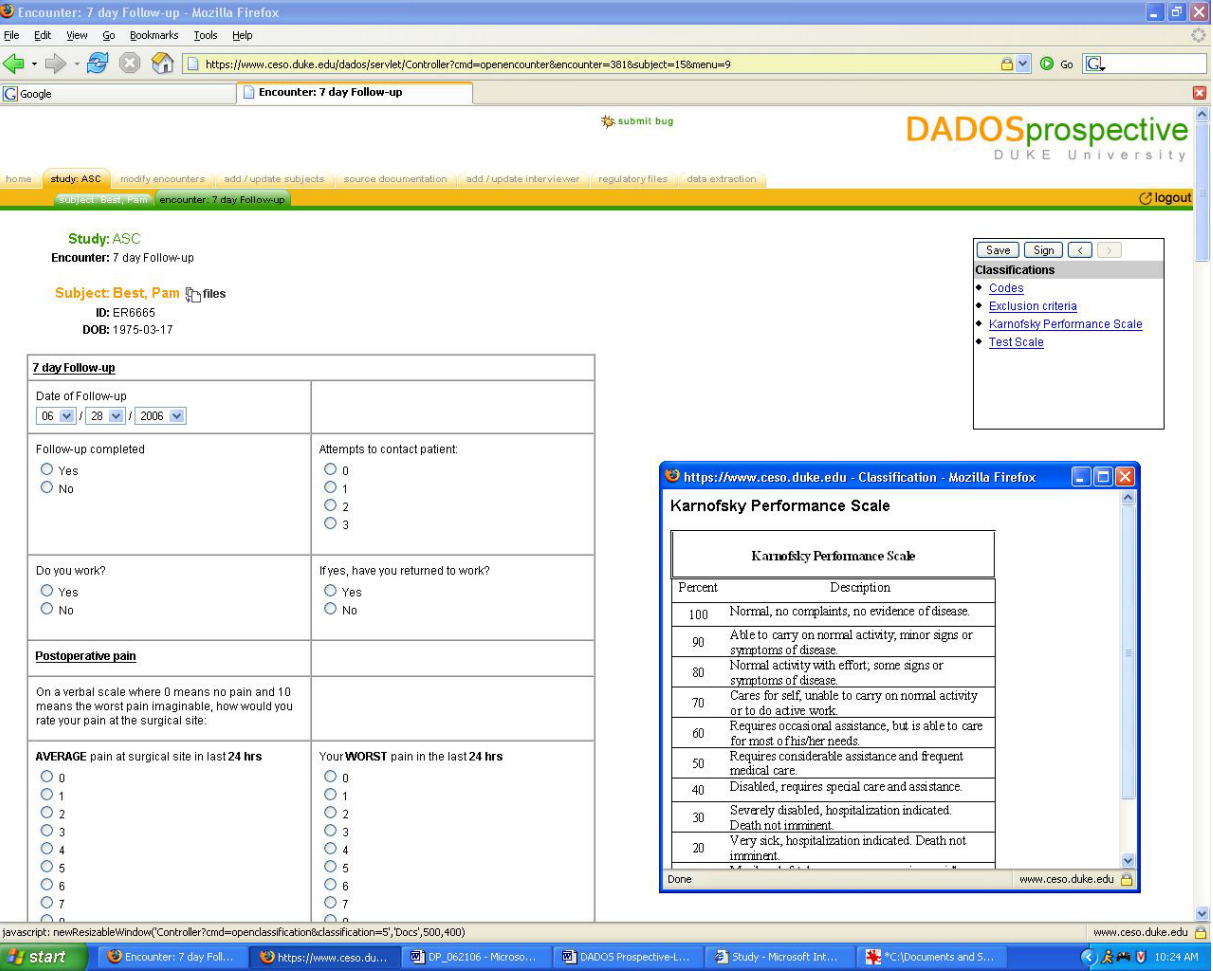
**Data entry**. Data entry interface demonstrating floating window of selected source documents.

Coordinators enroll patients into their study by using the "add/update subjects" tab in the toolbar. Selecting a patient's name brings forth the list of encounters that have been previously defined and the date on which the encounters are to take place [see Figure 5]. As mentioned earlier, this date is automatically calculated using the program's baseline function; however, this date may be manually set or modified by clicking on the calendar icon displayed next to the date. Should the coordinators wish to upload files for an encounter, such as uploading magnetic resonance images for the "Radiographic Diagnosis" encounter, they may do so using the icons under the "Files" heading [see Figure 5]. Data for the selected patient may be entered by clicking on each encounter and filling out the customized form and saving the information or signing it using the floating window buttons. The coordinators may perform the data entry themselves, or they assign the task to an "interviewer," whose access to the study is strictly limited to data entry. Interviewers may be added or removed by utilizing the "Add/Update Interviewer" function in the navigation toolbar. Additionally, in an effort to facilitate data entry, DADOS-Prospective allows the coordinator to upload any reference/source document (i.e. inclusion/exclusion criteria, rating scales, Web links, image files, etc.) relevant to the study onto the application where it can be instantly accessed by an interviewer. As described earlier, these documents can be uploaded and accessed by selecting the "source document" tab on the navigation toolbar. Once uploaded, a coordinator or an interviewer may select the desired reference documents to be displayed in a floating window that appears on the screen as they fill out data forms [see Figure 6]. This feature is highly convenient during instances when the interviewer/coordinator may feel compelled to double check criteria and/or grading scales in order to accurately enter data into the form. Similarly, the coordinator can also add and access regulatory files, such as IRB related materials, study procedure protocol and laboratory certifications, onto the program by utilizing the "regulatory files" tab function.

**Figure 7:**
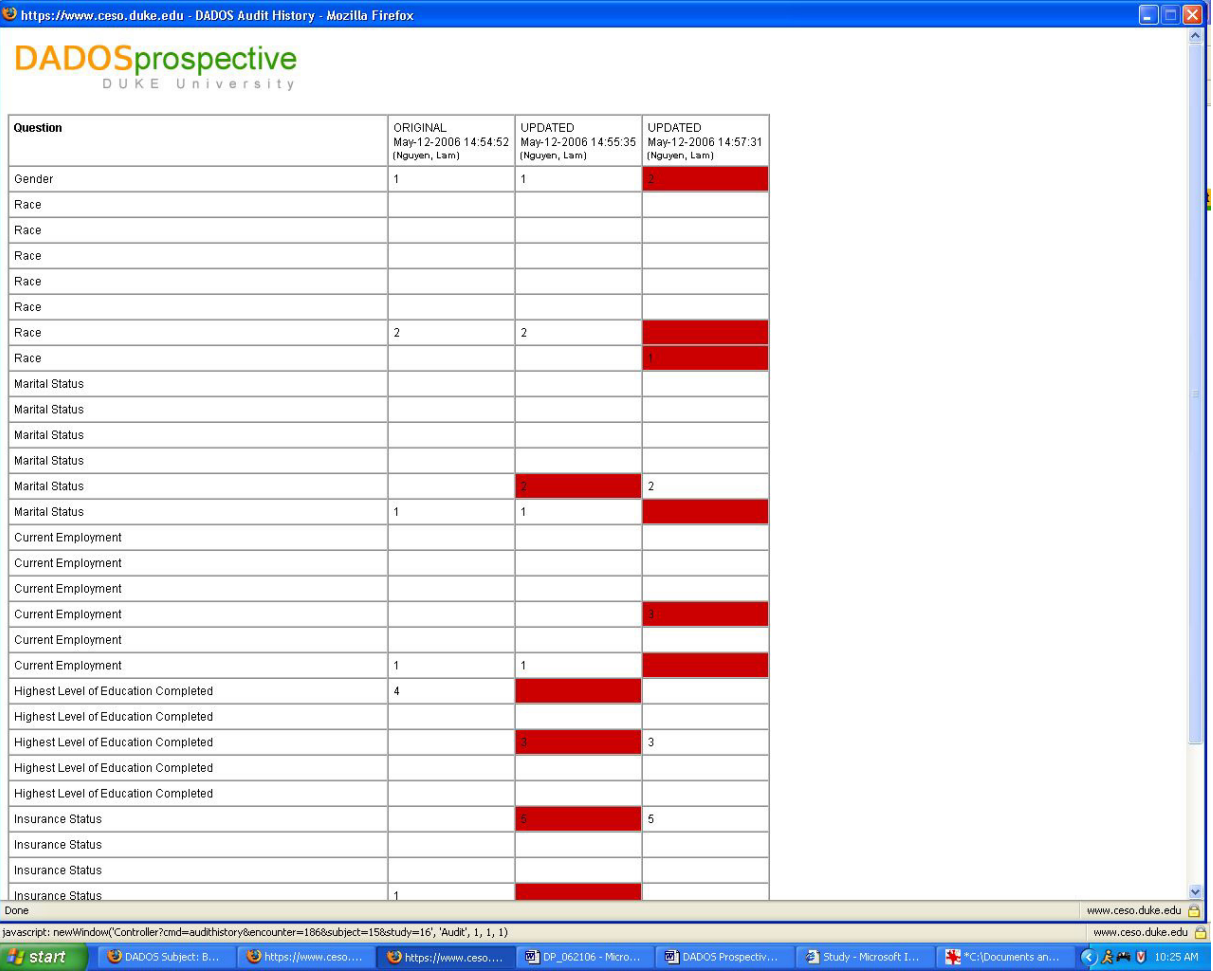
**Audit trail**. Audit trail interface

DADOS-Prospective contains an audit trail capability that enables PI's and coordinators to track changes each time data in a form is edited. This feature is crucial for maintaining data quality, especially when the tasks of data entry and data quality control are handled by two different parties. An "audit trail" link is displayed adjacent to each data form, thus improving the visibility for data quality control and making data auditing easy and simple [see Figure 5]. Clicking on the link, the user will see the CRF with fields that have recently been edited highlighted in red, showing the latest entry and all previous entries plus date and time of modification [see Figure 7].

As data is entered onto the forms, it is automatically stored in password-protected, encrypted servers at the host institution. At any time during the data collection process, only an administrator can extract the data for analysis by using the tab labeled "Data extraction" in the navigation toolbar. The program stores data in a spreadsheet format, making it readily available for analysis upon extraction.

## Results

### Usability

To date, DADOS-Prospective has been implemented in five different studies conducted by various departments within the Duke University Health System. Four of these studies are still in the form creation phase, and one study has been officially launched. Our initial experiences have indicated that DADOS-Prospective has been used with relative ease by the investigators and the software has streamlined their study creation and management processes.

### Formal usability

Usability testing was performed in accordance with previous novel software applications from our institution [6]. Formal usability tests were performed using 10 physician-investigators who were computer literate by self-report. Each investigator was given a brief, 30 minute tutorial on DADOS-Prospective with an explanation of its purpose and basic functionality. Users were given 45 minutes to then explore the program on their own and complete a questionnaire that is summarized in Table 2. Formal usability results demonstrated that, from the perspective of researchers, DADOS-Prospective application presented good speed (100 percent strongly agreed), was easy to learn and use, had a functionality that was easily understandable, and a navigation that was intuitive [see Table 2]. No further features were requested during the formal usability analysis phase.

**Table 2:** Formal usability. Results from formal usability tests.

DADOS-Prospective speed is excellent.	Strongly disagree	0/10
	Disagree	0/10
	Neutral	0/10
	Agree	0/10
	Strongly agree	10/10
		
DADOS-Prospective is extremely easy to learn	Strongly disagree	0/10
	Disagree	0/10
	Neutral	0/10
	Agree	2/10
	Strongly agree	8/10
		
DADOS-Prospective is extremely easy to use	Strongly disagree	0/10
	Disagree	0/10
	Neutral	1/10
	Agree	2/10
	Strongly agree	7/10
		
It is very easy to understand all functionality available within DADOS-Prospective	Strongly disagree	0/10
	Disagree	0/10
	Neutral	0/10
	Agree	2/10
	Strongly agree	8/10
		
The navigation in DADOS-Prospective is highly intuitive	Strongly disagree	0/10
	Disagree	0/10
	Neutral	2/10
	Agree	1/10
	Strongly agree	7/10

### Field usability

The first three months of field usability measurement were focused primarily on fixing minor software problems related to coding, including buttons that were not working appropriately, navigational issues related to users being taken to the wrong page after completing a task, and the implementation of a consistent interface across all pages of the application. After initial field testing was completed, the system was tested with actual CRFs from various departments and tested with mock patient data. Additional navigational problems were identified and several features were requested to improve usability. Requested features included: navigational issues, additional form creation functions, addition of a function for "subject search," and additional data extraction methods.

### Current utilization

The DADOS-Prospective application is currently being used in five different studies. Four of these studies are still in the CRF creation phase and one study has been entirely launched. The active study in DADOS-Prospective replaced a pre-existing database process. The database is a registry for subjects who will undergo outpatient surgery (approximately 40 subjects per day), and houses the database that is used for retrospective studies for the Department of Anesthesiology. Implementation for this project included creating special plug-ins for the CRF which would allow for surgical procedures to be added to a list on the data collection form. This enables alternatives to be added and/or updated on the CRF when the plug-in is used for the purposes of form creation, even after the study has gone live, thus maintaining the integrity of the data collection and recognizing the ever-changing possibilities for surgical procedures and techniques. Feedback from data entry specialists indicates that they prefer the new database using DADOS-Prospective compared to the old database, adding that it is much easier to use and their process has been significantly streamlined.

## Discussion

Numerous programs have emerged to take advantage of the Internet as a primary means for collecting data in randomized, prospective clinical trials [1,7-11]. However, many of them are expensive, difficult to use, or they are only tailored to specific study designs. For example, commercial Windows-based software applications are limited in their ability to centralize data for file sharing in multi-institutional studies, to comply with HIPAA regulations in order to support electronic signatures, and to maintain an audit trail of use [12]. While commercial products do not permit modification of source code, we are confident that the modifiable source code of DADOS-Prospective will encourage users to utilize and improve upon the features in this release. Furthermore, unlike DADOS-Prospective, many existing open-source applications either do not have the flexibility to allow users to simultaneously customize study designs and CRFs for multiple clinical studies covering different medical subspecialties within the same software, or do not support user autonomy that allows individual users to create future studies at anytime (Web-based Electronic Patient Record System, Berlin, Germany; CARE Application, Hershey, PA; WARG study, Stockholm, Sweden; Delphi Method, New Heaven, CT).

We developed DADOS-Prospective as an open-source program that not only addresses the limitations of existing commercial platforms, but also possesses the versatility to create and manage any type of prospective clinical study. As such, the program is fully customizable by the institutional user. In addition to achieving full compliance with HIPAA regulations, the ability of the program to create virtually any CRF format allows investigators to use DADOS-Prospective to develop studies in any medical specialty. Furthermore, by possessing the capability to support future interview scheduling as well as a patient database monitoring system, DADOS-Prospective steps beyond the task of data collection and management and acts as a complete study management system. Most notably, the program contains in one single location source documents, regulatory files, raw data in spreadsheet format, audit trails, interview scheduling, patient database, and CRFs. This feature greatly enhances the visibility of the two crucial aspects of coordination and management of studies and data, thereby making DADOS-Prospective an ideal tool in conducting prospective clinical trials. More crucially, due to the fact that it was designed to work under slow Internet data transfer speeds, DADOS-Prospective is especially suitable for multicenter prospective international studies, thus taking advantage of a large population base. One limitation we have discovered is the lack of communication channels between users within the software application; this is a function that we feel would greatly enhance the management of the multicenter studies. We are currently developing potential solutions for this issue.

Whereas previous Web-based prospective clinical studies may have taken up to nine months to develop using existing tools, our initial experiences have demonstrated that complex studies were developed within 3–4 months using DADOS-Prospective. The program's user-friendly interface makes DADOS-Prospective quite easy to use and does not require much training. Although several studies launched at our own institution had few minor issues with some features (saving follow-up dates, updating patient registration information, minor difficulties editing CRFs with certain versions of Microsoft Internet Explorer), these problems were quickly addressed and corrected. We have also recognized the need to constantly increase the security of our software and are currently seeking new ways to do this.

## Conclusion

We have demonstrated that the current version of DADOS-Prospective has significant advantages over existing Web-based data collecting programs. The program's ease of use, enhanced visibility for data auditing, versatility of CRF creation, simple data query mechanisms, and full compliance with HIPAA regulations make DADOS-Prospective an ideal tool for creating and managing prospective clinical trials.

## Availability and requirements

Project name: DADOS-Prospective

Project home page:  (click link for "Free software")

Operating systems: Linux and Windows

Programming language: Java

Other requirements: Tomcat 5.x

License: GNU General Public License. This license ensures that the source code can be freely distributed, modified, or even sold, as long as the source code is provided with every copy of the application. The source code for the application is available at no charge.

Any restrictions to use by non-academics: None.

## Abbreviations used

CRF: Clinical research form

HIPAA: Health Insurance Portability and Accountability Act

PI: Principal investigator

MVC: Model-View-Controller

## Competing interests

The author(s) declared that they have no competing interests.

## Authors' contributions

All authors have read and approved the final manuscript. LN assisted in the design of the application, field tests for detection of bugs, design and analysis of usability tests, and manuscript draft. AS assisted in the design of the application, field tests for bug detection, and revision of manuscript draft. MH assisted in the design of the application, field tests for bug detection, and revision of manuscript draft. HM assisted in the design of the application, wrote source code, and revised manuscript draft. MM assisted in the design of the application, field tests for bug detection, design and analysis of usability tests, and revised manuscript draft. AM assisted in the design of the application, field tests for bug detection, and revised manuscript draft. DJ assisted in the design of the application, field tests for bug detection, and revision of manuscript draft. RP assisted in the design of the application, field tests for detection of bugs, design and analysis of usability tests, and manuscript draft.

## References

[B1] Lallas CD, Preminger GM, Pearle MS, Leveillee RJ, Lingeman JE, Schwope JP, Pietrow PK, Auge BK (2004). Internet based multi-institutional clinical research: a convenient and secure option. J Urol.

[B2] Lopez-Carrero C, Arriaza E, Bolanos E, Ciudad A, Municio M, Ramos J, Hesen W (2005). Internet in clinical research based on a pilot experience. Contemp Clin Trials.

[B3] Cooper CJ, Cooper SP, Del Junco DJ, Shipp EM, Whitworth R, Cooper SR (2006). Web-based data collection: detailed methods of a questionnaire and data gathering tool. Epidemiol Perspect Innov.

[B4] 21 CFR Part 11: Electronic Records; Electronic Signatures  [http://www.fda.gov/ora/compliance_ref/part11/].

[B5] Pietrobon R, Shah A, Kuo P, Harker M, McCready M, Butler C, Martins H, Moorman CT, Jacobs DO (2006). Duke Surgery Research Central: an open-source Web application for the improvement of compliance with research regulation. BMC Med Inform Decis Mak.

[B6] Shah A, Jacobs DO, Martins H, Harker M, Menezes A, McCready M, Pietrobon R (2006). DADOS-Survey: an open-source application for CHERRIES-compliant Web surveys. BMC Med Inform Decis Mak.

[B7] Winget M, Kincaid H, Lin P, Li L, Kelly S, Thornquist M (2005). A web-based system for managing and co-ordinating multiple multisite studies. Clin Trials.

[B8] Lindh JD, Kublickas M, Westgren M, Rane A (2004). Internet based clinical trial protocols -- as applied to a study of warfarin pharmacogenetics. Br J Clin Pharmacol.

[B9] Schmidt JR, Vignati AJ, Pogash RM, Simmons VA, Evans RL (2005). Web-based distributed data management in the childhood asthma research and education (CARE) network. Clin Trials.

[B10] Fritsche L SK (2000). A Web-based Electronic Patient Record System as a Means for Collection of Clinical Data.. ISMDA.

[B11] Deshpande AM, Shiffman RN, Nadkarni PM (2005). Metadata-driven Delphi rating on the Internet. Comput Methods Programs Biomed.

[B12] Kline JA, Johnson CL, Webb WB, Runyon MS (2004). Prospective study of clinician-entered research data in the Emergency Department using an Internet-based system after the HIPAA Privacy Rule. BMC Med Inform Decis Mak.

